# Kinetic modulation of bacterial hydrolases by microbial community structure in coastal waters

**DOI:** 10.1111/1462-2920.16297

**Published:** 2022-12-19

**Authors:** Naiara Abad, Ainhoa Uranga, Begoña Ayo, Jesús Maria Arrieta, Zuriñe Baña, Iñigo Azúa, Itxaso Artolozaga, Juan Iriberri, Santos J. González‐Rojí, Marian Unanue

**Affiliations:** ^1^ Department of Immunology, Microbiology and Parasitology, Faculty of Science and Technology University of Basque Country (UPV/EHU) Leioa Spain; ^2^ Department of Zoology and Animal Cell Biology, Faculty of Pharmacy University of the Basque Country (UPV/EHU) Alava Spain; ^3^ Research Centre for Experimental Marine Biology and Biotechnology PiE‐UPV/EHU Plentzia Spain; ^4^ Canary Islands Oceanographic Center Spanish Institute of Oceanography (IEO‐CSIC) Santa Cruz Spain; ^5^ Oeschger Centre for Climate Change Research (OCCR) University of Bern Bern Switzerland; ^6^ Climate and Environmental Physics (CEP) University of Bern Bern Switzerland

## Abstract

In this study, we hypothesized that shifts in the kinetic parameters of extracellular hydrolytic enzymes may occur as a consequence of seasonal environmental disturbances and would reflect the level of adaptation of the bacterial community to the organic matter of the ecosystem. We measured the activities of enzymes that play a key role in the bacterial growth (leucine aminopeptidase, β‐ and α‐glucosidases) in surface coastal waters of the Eastern Cantabrian Sea and determined their kinetic parameters by computing kinetic models of distinct complexity. Our results revealed the existence of two clearly distinct enzymatic systems operating at different substrate concentrations: a high‐affinity system prevailing at low substrate concentrations and a low‐affinity system characteristic of high substrate concentrations. These findings could be the result of distinct functional bacterial assemblages growing concurrently under sharp gradients of high‐molecular‐weight compounds. We constructed an ecological network based on contemporaneous and time‐delayed correlations to explore the associations between the kinetic parameters and the environmental variables. The analysis revealed that the recurring phytoplankton *blooms* registered throughout the seasonal cycle trigger the wax and wane of those members of the bacterial community able to synthesize and secrete specific enzymes.

## INTRODUCTION

Heterotrophic bacteria, through the synthesis and release of extracellular enzymes, are the main transformers of the high‐molecular‐weight dissolved organic matter (HMW‐DOM) into low‐molecular‐weight compounds (<600 Da) (Arnosti, 2003; Chróst, 1991), which are therefore available for microbial uptake (Decad & Nikaido, 1976; Weiss et al., 1991) and the subsequent production of biomass and energy. Thus, extracellular enzymatic activities (EEAs) are considered the rate limiting step in the mineralization of organic matter and therefore, in the transference of organic matter to higher trophic levels in marine systems (Chróst, 1991).

EEAs in aquatic ecosystems have been extensively determined using both fluorescent substrate proxies and fluorescently labelled substrates (Hoppe, 1983; Pantoja et al., 1997). Likewise, hydrolysis rates of EEAs at a single substrate saturating concentration have been extensively studied in field observations (Baña et al., 2020; Celussi & Negro, 2012), microcosm/mesocosm experiments (Alldredge et al., 1995; Maßmig et al., 2019; Unanue et al., 1998) and throughout the water column (Baltar et al., 2009; Misic et al., 2006; van Wambeke et al., 2021).

In contrast, information about the kinetics of EEAs in coastal and open ocean waters is scarce and the available studies cover a short temporal scale, commonly less than a year of monitoring (Unanue et al., 1999; van Wambeke et al., 2021; Williams & Jochem, 2006). However, the determination of kinetic parameters of EEAs under different environmental situations is of great interest because it allows to make a direct connection between enzyme expression and availability of substrate (Sinsabaugh et al., 2014). The accessibility of economic and sensitive fluorescence plate readers makes these kinetic studies feasible, greatly reducing the amount of substrate used and the manipulation burden as compared to the cuvette measurements used in most of the available literature.

Another important issue when performing the determination of the kinetic parameters of the EEAs is the model used to fit the experimental data. The vast majority of the studies carried out in seawater ecosystems rely on the assumption that the enzymatic reactions obey the conventional Michaelis–Menten equation (Michaelis & Menten, 1913) both when the kinetic parameters were calculated by Hanes–Woolf or Lineweaver–Burk linearisation methods and with non‐linear regression models (Maßmig et al., 2019; Urvoy et al., 2020; Williams & Jochem, 2006). On the contrary, complex models have been neglected, with few exceptions (Talbot & Bianchi, 1997; Tholosan et al., 1999; Unanue et al., 1999; van Wambeke et al., 2021; Vrba et al., 1996).

In the euphotic zone, there are diverse production mechanisms of DOM, among which the direct excretion by phytoplankton photosynthesizing cells or during death processes represents the most important carbon source for the bacterial community (Moran et al., 2022; Myklestad, 2000; Thornton, 2014). Giving the heterogeneous nature of DOM and considering that each of the species conforming the natural bacterial assemblages may display a combination of different enzymes acting towards a substrate (Arrieta & Herndl, 2002; Vrba et al., 1996; Williams, 1973), it is highly controversial to assume that kinetics of natural EEAs are well characterized by a simple Michaelis–Menten model. Consequently, more complex equations should also be included for the determination of kinetic parameters of the EEAs in natural environments.

The goal of the present study is twofold. First, to determine the mathematical model that most accurately describes the kinetic curves of leucine aminopeptidase (LAP), β‐glucosidase (βG) and α‐glucosidase (αG) activities in surface seawaters of a temperate ecosystem. And second, to examine the relationship between transient pulses of organic matter related to phytoplankton *blooms* and/or shifts in the bacterial community composition and the EEAs observed in the field.

## EXPERIMENTAL PROCEDURES

### Sampling strategy

Monthly sampling was conducted at 9.00 am ± 30 min local time at Armintza Station (43°26′2.68″N, 2°54′2.21″W), located in the southeastern part of the Bay of Biscay. In total, 27 samples were collected from February 2011 to September 2013. Surface seawater was collected in an acid‐washed (1% HCl) 10‐L polyethylene bucket thoroughly rinsed with Milli‐Q water. Samples were pre‐filtered through a 100‐μm nylon mesh to exclude large planktonic organisms. Surface seawater temperature and salinity were measured in situ with a calibrated probe (VWR EC300).

### Extracellular enzymatic activities

The samples used for enzymatic assays were rapidly transferred to the laboratory in precombusted opaque flasks (400°C, 4 h). A set of extracellular enzyme activities was assayed using fluorogenic substrate analogues according to Hoppe (1983). This method is based on the addition of artificial substrate proxies (Arrieta & Herndl, 2002; Hoppe, 1983; Steen et al., 2015); therefore, the results presented in this study should be interpreted as an approximation to the hydrolysis rates of naturally occurring substrates. The substrate proxies were l‐leucine‐7‐amide‐4‐methylcoumarin hydrochloride, 4‐MUF‐β‐d‐glucoside, and 4‐MUF‐α‐d‐glucoside to estimate the hydrolysis rates of LAP, βG and αG, respectively. Solutions containing different concentrations of 7‐amino‐4‐methylcoumarinyl (MCA) or 4‐methylumbelliferone (MUF) were used as standards to allow conversion of fluorescence readings into concentrations of the corresponding end product. Stock solutions of reagents were prepared by dissolving the crystalline form in absolute methanol to facilitate solubilization and then adding Milli‐Q water (40% methanol final concentration). The reagents were stored at −20°C until use.

Working solutions of substrates were prepared in a microplate by diluting the stock solutions in Milli‐Q water in serial half‐dilutions. Ten microliters of each dilution were added to 240 μl seawater samples in order to prepare 12 different final experimental concentrations between 0 and 400 μM for LAP and 0–300 μM for the glucosidases. Four replicates were prepared for each concentration and duplicate sample blanks (240 μl seawater + 10 μl Milli‐Q water) were used to determine the background fluorescence of the samples. Enzymatic assays were conducted in black 96‐well microplates (Nunc) incubated at in situ temperature. Fluorescence readings were obtained at 365 nm excitation/445 nm emission wavelengths using a commercial fluorescence plate reader (Synergy 2, Biotek). The increase in fluorescence over time was transformed to concentrations the corresponding end product by using standard curves of MCA (final concentrations in the range from 0 to 1000 nM) and MUF (final concentrations in the range from 0 to 500 nM). The rates of enzymatic activity (nmol·L^−1^·h^−1^) were calculated by dividing the increase in the concentration of the corresponding end product (MUF or MCA) by the incubation time. Incubation times from 3 to 6 h were enough to obtain a significant increase in fluorescence. Previous experiments showed that abiotic hydrolysis of the substrate was negligible. LAP was not measured in July 2013.

### Determination of the kinetic parameters

For each enzymatic activity and sample, the determination of the kinetic parameters was addressed by fitting the rates of enzymatic activity to four different kinetic models (Figure S1) of increasing complexity (Vrba et al., 1996) using a non‐linear least squares regression, as implemented in the *nls* function included in the *stats* package of R software (version 3.6.1) (R Core Team, 2019).

**Model 1:** Represents a single enzyme system with first‐order kinetics indicating that the range of concentrations tested did not approach saturation. Under these conditions it is not possible to estimate *V*_max_ and *K*_m_ but the model can be formulated as:

$$V=T_{t}\cdot S,$$

where *V* is the velocity of hydrolysis of the reaction, *T*_t_ is the turnover time (equivalent to the ratio *V*_max_/*K*_m_), and *S* is the substrate concentration.

**Model 2:** Represents a single enzyme system following the classic Michaelis–Menten model (Michaelis & Menten, 1913):

$$V=\frac{V_{\max}\cdot S}{K_{m}+S},$$

where *V*_max_ is the maximum hydrolysis rate of the enzyme reaction obtained at a saturating concentration of substrate and *K*_m_ is the Michaelis half‐saturation constant indicating the concentration of substrate needed to obtain half of *V*_max_.

**Model 3:** Represents a two‐enzyme system, whose kinetics are the sum of two groups of independent isoenzymes. A high‐affinity (HA) system saturated at low substrate concentrations, and therefore described by Model 2, and a low‐affinity (LA) system not reaching saturation within the range of concentrations tested and thus, best described by Model 1:

$$V=\frac{V_{\max\;\mathrm{HA}}\cdot S}{K_{m\;\mathrm{HA}}+S}+T_{t\;\mathrm{LA}}\cdot S.$$

**Model 4:** Represents a two‐enzyme system, with two groups of independent isoenzymes approaching saturation within the range of substrate concentrations tested but showing markedly different kinetic parameters. The subscripts HA and LA stand for the high‐affinity and low‐affinity enzymatic systems, respectively:

$$V=\frac{V_{\max\;\mathrm{HA}}\cdot S}{K_{m\;\mathrm{HA}}+S}+\frac{V_{\max\;\mathrm{LA}}\cdot S}{K_{m\;\mathrm{LA}}+S}.$$

The model best representing field data was chosen based on the corrected Akaike's information criteria (AICc), since the *F*‐test has a strong tendency to choose the simpler model when the competing models are similar, even when the more complex one is correct (Glatting et al., 2007; Ludden et al., 1994). A more complex model was accepted when it improved the fit based on a lower AICc value.

Cell‐specific maximum hydrolysis rates (sp. *V*_max_) were calculated by dividing the *V*_max_ of each sample by the bacterial abundance observed in the samples (see below).

Differences between the kinetic parameters of HA and LA systems were tested using the non‐parametric Wilcoxon signed rank test for paired‐samples (*p* ≤ 0.05). The statistical analysis was performed with *IBM SPSS Statistics* (version 24) for Windows.

### Bacterial abundance

Bacterial abundance (cells·L^−1^) was determined by epifluorescence microscopy following the protocol established by Porter and Feig (Porter & Feig, 1980). For each sample, 10 ml of seawater aliquots was fixed with 0.2 μm‐filtered, borate‐buffered formalin (2% v/v final) immediately after sampling and stored at 4°C in the dark until processed. Subsamples of 1 ml were stained with 4′,6‐diamidino‐2‐phenylindole (DAPI; 0.02 mg·ml^−1^ final concentration) for 10 min. The stained cells were then filtered onto 0.22‐μm pore‐sized black polycarbonate filters (Whatman) and examined using a Nikon Optiphot epifluorescence microscope by direct counting of randomly selected microscopic fields. Microscope slides were prepared and counted within a few hours of sampling.

### Chlorophyll *a*

Daily average chlorophyll *a* concentration (μg·L^−1^) was obtained from AQUA‐MODIS satellite data (https://oceancolor.gsfc.nasa.gov/l3/). Level 3 (L3) files with a 4‐km spatial resolution were retrieved and processed by using *RNetCDF* package (Michna & Woods, 2013) of R software (Version 3.6.1) (R Core Team, 2019).

### Cyanobacteria

Marine cyanobacteria were enumerated by flow cytometry following the protocol described in Marie et al. (Marie et al., 1999). Unfixed samples were analysed on a FACSCalibur flow cytometer (Becton‐Dickinson) equipped with blue laser emitting at 488 nm and were run at high speed for 10 min or until reaching 100,000 events. Different populations were classified as *Synechococcus* and *Prochlorococcus* by their size and pigment content based on the intensity of side scattered light and the fluorescence emission in the orange (585/42 nm BP filter) and red (670 nm LP filter) wavelengths. The reported cyanobacterial abundances (cell·L^−1^) correspond to the sum of *Synechococcus* and *Prochlorococcus* cell abundances.

### Bacterial community composition

The phylogenetic affiliation of bacteria was determined by CARD‐FISH following the protocol described by Pernthaler et al. (Pernthaler et al., 2002). For each sample, 25 ml of seawater aliquots were fixed with paraformaldehyde (2% v/v final concentration) and stored at 4°C in the dark overnight. Samples were then filtered through 0.22‐μm pore‐sized polycarbonate filters (Millipore GTTP). The cells deposited on the filter were permeabilized with lysozyme (37°C, 1 h) prior to hybridization at 35°C for 2 h in the presence of the corresponding probe. Following amplification, a fluorescent signal was developed tyramide‐Alexa488 amplification for 15 min. Horseradish peroxidase‐labelled probes were added to specifically target: the domain *Bacteria* (EUB I; Amann et al., 1990) plus the complementary EUB II and EUB III probes targeting *Planctomicetales* and *Verrucomicrobiales*, respectively, which are not detected by EUB I (Daims et al., 1999). In addition, we used group‐specific probes targeting major groups of marine bacteria like SAR11 (SAR11‐441R; Morris et al., 2002), members of the *Roseobacter* and SAR83 clades (ROS537; Eilers et al., 2001), *Gammaproteobacteria* (Gam42a; Manz et al., 1992) and *Bacteroidetes* (CF319a; Manz et al., 1996). The antisense control probe NON338 (Wallner et al., 1993) was used as a negative control for non‐specific binding. Formamide concentration in the hybridization buffer was 55% for all probes except for the NON338 control probe, for which only 20% formamide was used. Filter sections were DAPI stained after hybridization before they were counted under the epifluorescence microscope. For each sample, the relative abundance of the different phylogenetic groups was calculated relative to the total bacterial abundance.

### Local similarity analysis

Shifts in the microbial community structure or induction of enzymatic activities can be very fast, but often show a certain delay as compared to the primary *driver*. For example, a phytoplankton *bloom* may cause an increase in the availability of substrates, but this increase will be available to bacteria only after the *bloom* has collapsed (Becker et al., 2014; Bidle & Azam, 2001; Myklestad, 2000) resulting in no apparent correlation between an increase in enzymatic activity and the primary causative agent (phytoplankton *bloom*). We used a modified version (see *Data availability*) of the local similarity analysis (LSA) procedure (Ruan et al., 2006) allowing up to 1 month delay in order to detect contemporary and time‐delayed correlations between primary producers, major bacterial groups and the expression the different enzymatic activities. The results were represented as a network by using Cytoscape 3.7.2 (Shannon et al., 2003) in order to obtain an integrated view of the complex dynamics that occur in our study site.

## RESULTS

### Determination of the kinetic parameters

**FIGURE 1:**
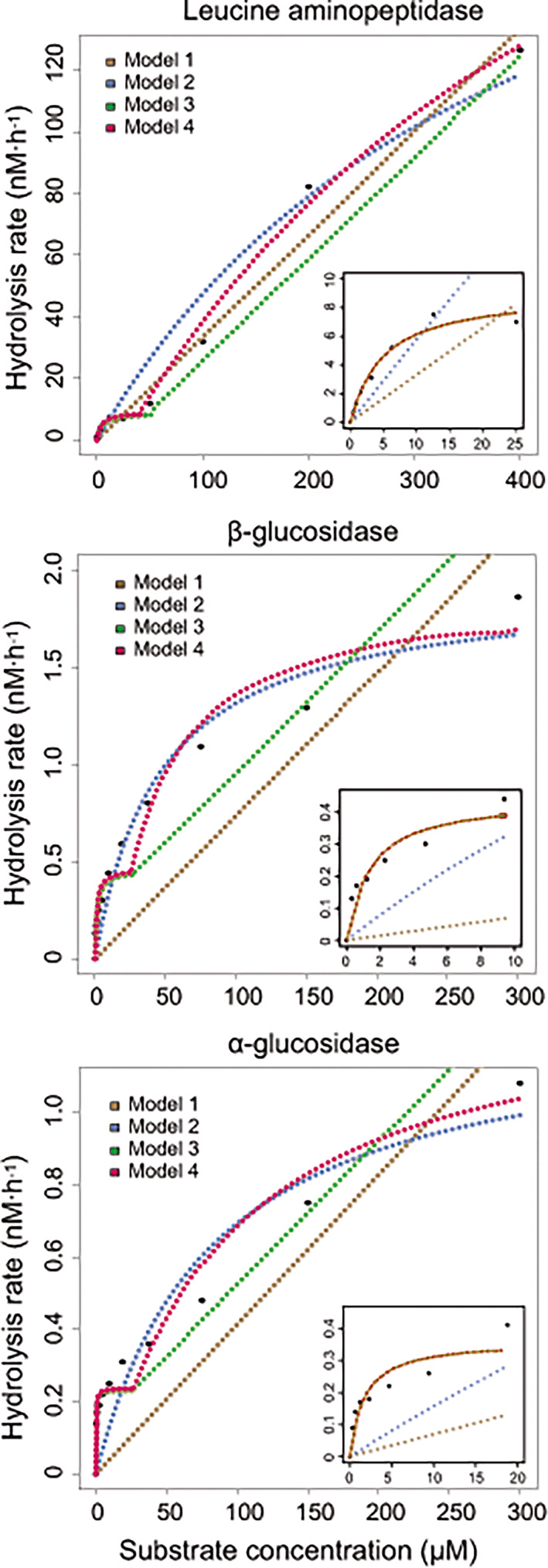
Plots of the hydrolysis rates of the three enzymatic activities in sample February 2012 of Armintza station. Black circles represent the response curve for the whole assayed range of substrate concentrations in the enzymatic assay and the dashed lines represent the different mathematical models computed. Insets correspond to the fit of the models to data at low substrate concentrations.

Field data corresponding to LAP, βG and αG were fitted to four different models of increasing complexity to determine the kinetic parameters as described in Experimental procedures (see Figure 1). According to the AICc, Model 4 best described the kinetics of LAP in all the seawater samples analysed and in 94% of the samples in the case of the glucosidases (Table S1). The only exceptions were samples October 2011 and November 2011 for βG, where only one, LA system was detectable (Model 2), and the sample collected in November 2011 for αG, which was best described by Model 3 (Table S1). Thus, the response curves of all the EEAs revealed the existence of at least two different enzymatic systems showing marked differences in both their affinity for the substrate and the maximum hydrolysis rate. These two enzymatic systems could be detected throughout the year with few exceptions for the almost three‐year duration of the study.

**FIGURE 2:**
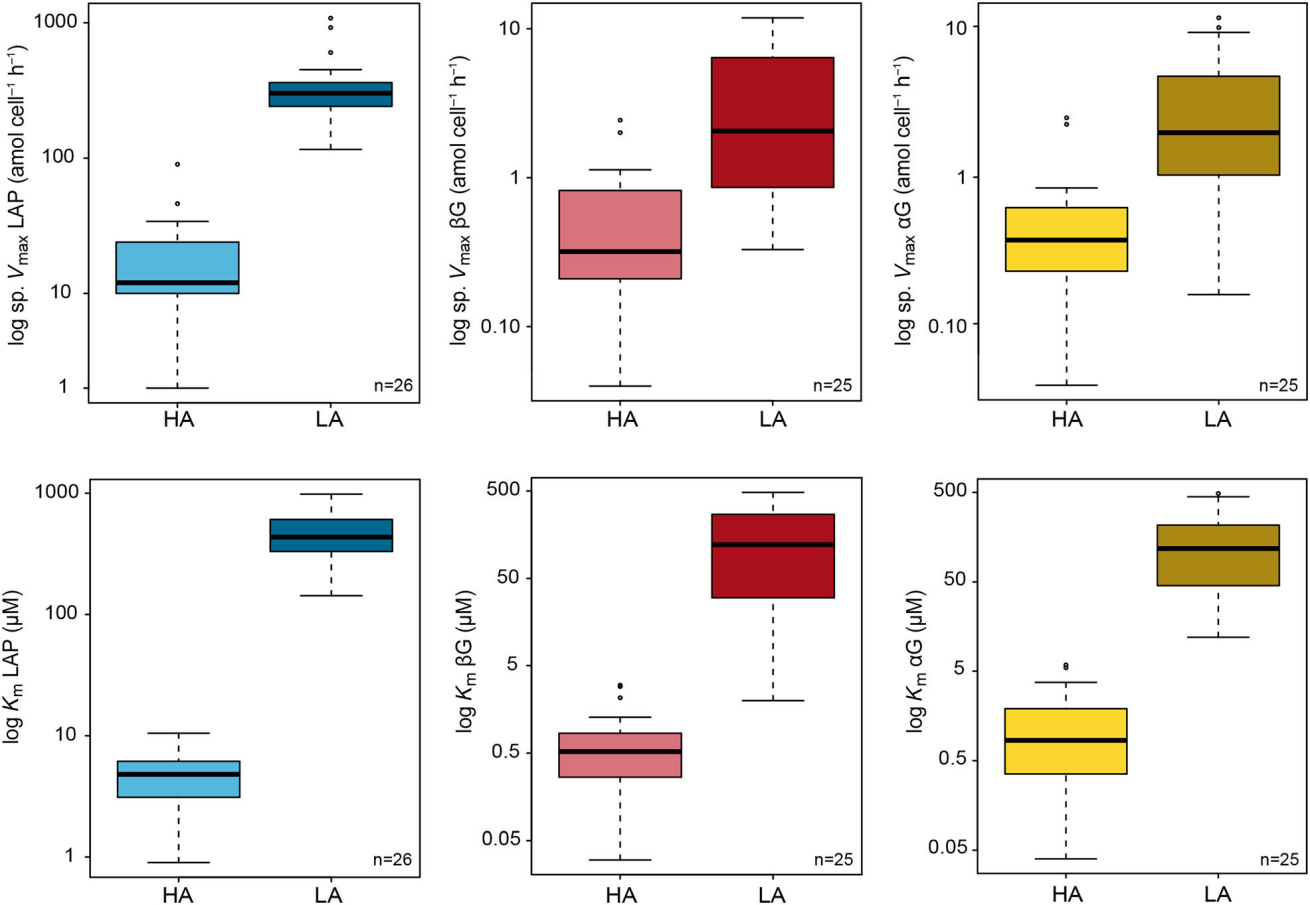
Box–whisker plots of the kinetic parameters cell‐specific maximum hydrolysis rate (sp. *V*_max_, top) and Michaelis half‐saturation constant (*K*_m_, bottom) of the three extracellular enzymatic activities: leucine aminopeptidase (LAP, blue), β‐glucosidase (βG, red), and α‐glucosidase (αG, yellow). Kinetic parameters are distinguished in light colour for the high‐affinity (HA) system and dark colour for the low‐affinity (LA) system. In all the cases, statistically significant differences were found between the two enzymatic systems (Wilcoxon signed‐rank test for paired samples, *p* ≤ 0.01).

The potential bulk activity (*V*_max_) of the LA enzymes was always about one order of magnitude higher than that of their HA counterparts (Figure 2 and Tables S2 and S3).

Two different LAPs were readily detectable in all samples throughout the study, a HA system characterized by low cell‐specific maximum hydrolysis rates and low *K*_m_ values (sp. *V*_max HA_: 2–90 amol·cell^−1^·h^−1^ and *K*_m HA_: 0.9–11 μM) and a LA system clearly distinguished by high values of the kinetic parameters (sp. *V*_max LA_: 116–1083 amol·cell^−1^·h^−1^ and *K*_m LA_: 143–983 μM) (Wilcoxon test for paired samples, *n* = 26 and *p* ≤ 0.01 in all the cases). It should be noted that in some samples the values obtained for the LA system were above the range of substrate concentrations used in the kinetic assays.

In a similar way, the HA systems of the glucosidases were characterized by low cell‐specific maximum hydrolysis rates and low *K*_m_ values (sp. *V*_max HA_: 0.03–2.40 amol·cell^−1^·h^−1^ and *K*_m HA_: 0.03–6 μM), whereas the LA system values differed significantly by orders of magnitude (sp. *V*_max LA_: 0.1–11.8 amol·cell^−1^·h^−1^ and *K*_m LA_: 2–486 μM) (Wilcoxon test for paired samples, *n* = 25 and *p* ≤ 0.01 in all the cases). Some estimates of the LA *K*_m_ exceeded the maximum substrate concentration added in the kinetic assay and encompassed the 30% (βG) and 15% (αG) of the total number of samples. In the three samples for which Models 2 and 3 better fitted the response curves of the hydrolysis rates, the estimates fell well within the range of values mentioned above.

### Seasonal dynamics of EEAs

**FIGURE 3:**
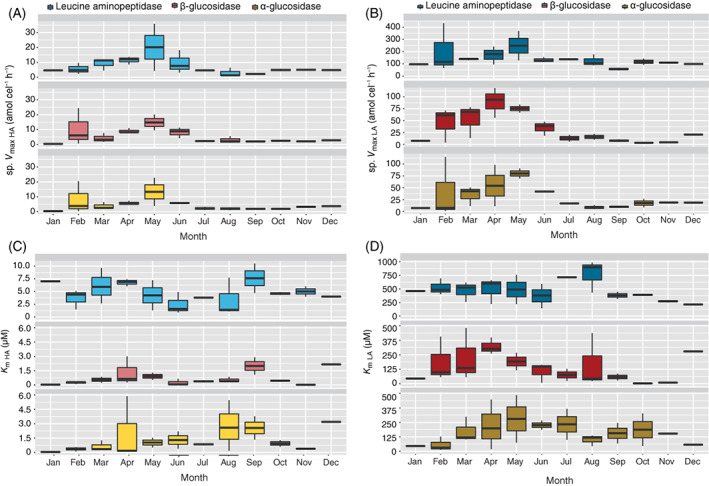
Box–whisker plots of the kinetic parameters of α‐glucosidase (αG, yellow), β‐glucosidase (βG, red) and leucine aminopeptidase (LAP, blue). Experimental data of the cell‐specific maximum hydrolysis rate (sp. *V*_max_) are represented in the upper panels and the half‐saturation constant (*K*_m_) below. The subscripts HA and LA stand for the high‐affinity (left) and low‐affinity (right) enzymatic systems. Note the different scales for LAP and glucosidases.

The specific *V*_max_ of the three enzymatic activities displayed a similar trend for the HA and LA systems (Figure 3A,B). Overall, specific *V*_max_ increased between February and April–May, when it reached its maximum value, followed by a sharp decline that continued until the end of the year. The tendency of the *K*_m_ was more variable. *K*_m_ values of the HA system increased between February and April and peaked in late summer for the three enzymatic activities (Figure 3C). The LA system showed high *K*_m_ values in spring and summer with lower values in winter, but summertime peaks were more pronounced in the case of LAP activity (Figure 3D).

**FIGURE 4:**
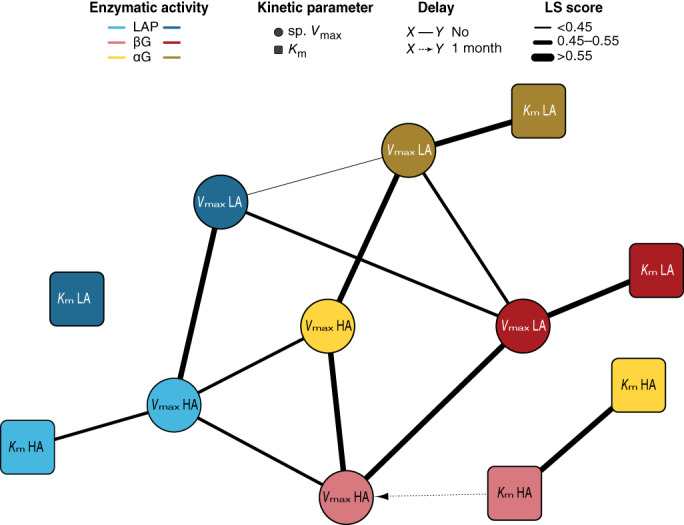
Network map of the positive intraspecific associations of the kinetic parameters of the three extracellular enzymatic activities. The nodes indicate the enzymatic activities leucine aminopeptidase (LAP, blue), β‐glucosidase (βG, red) and α‐glucosidase (αG, yellow). Kinetic parameters are distinguished in light colour for the high‐affinity (HA) system and dark colour for the low‐affinity (LA) system. Solid lines show a contemporary correlation and dashed lines with an arrow show a 1‐month shift in the correlation. The thickness of the lines differentiates the range of the correlation values (LS score).

The enzymatic association network deduced from the LSA is summarized in Figure 4 (Table S4). Within the same enzymatic system, we found an overall positive relationship between the sp. *V*_max_ and *K*_m_, except for the LA system of LAP and the HA system of αG. When we looked at the correlations between the kinetic parameters of the HA and LA systems, we observed a positive and contemporary correlation between the sp. *V*_max_ for each of the enzymatic activities. Indeed, this association pattern scaled up and a cluster between the sp. *V*_max_ of all the enzymatic activities was revealed, that was tighter between βG and αG activities as compared to LAP for both enzymatic systems. In contrast, no correlation was found between the values of *K*_m_.

### Associations with relevant drivers of the ecosystem

The analysis of chlorophyll *a* concentration and cyanobacterial abundance showed two recurring natural phytoplankton *bloom*s in this coastal ecosystem: an earlier one during spring dominated by eukaryotic members and another one in the late summer‐early autumn dominated by prokaryotic phytoplankton (Figure S2A,B). In the same way, it was detected an annually recurrent alternating dominance of *Bacteroidetes*, more abundant between April and late summer, and SAR11, more abundant between October and early spring (Figure S2C). *Gammaproteobacteria* generally exhibited a higher contribution during summer, whereas *Roseobacter* showed marked peaks of abundance between February and April. More detailed information about seasonal traits of the bacterial community in Armintza station can be found in Baña et al. (2020).

**FIGURE 5:**
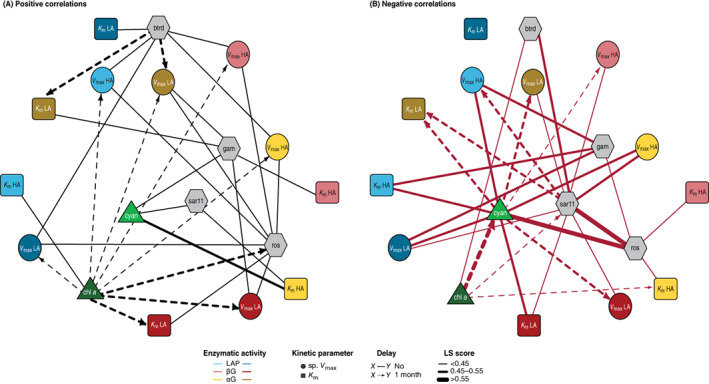
Network map of the associations between the kinetic parameters of extracellular enzymatic activities, the primary producers and the major groups of the bacterial community. Positive correlations (A) are represented on the left and negative correlations (B) are represented on the right. The nodes indicate the enzymatic activities leucine aminopeptidase (LAP, blue), β‐glucosidase (βG, red) and α‐glucosidase (αG, yellow), the primary producers (green) and the phylogenetic groups of the bacterial community (grey). Kinetic parameters are distinguished in light colour for the high‐affinity (HA) system and dark colour for the low‐affinity (LA) system. Solid lines show a contemporary correlation and dashed lines with an arrow show a 1‐month shift in the correlation. The thickness of the lines differentiates the range of the correlation values (LS score). btrd, *Bacteroidetes* (%); Chl *a*, Chlorophyll *a* concentration (μg·L^−1^); cyan, cyanobacterial abundance (cell·L^−1^); sar11, SAR11 (%); ros, *Roseobacter* and members of SAR83 (%).

When we investigated the association network with primary producers, the distribution of the positive and negative correlations was closely related to the main phytoplanktonic groups. Our results unveiled a 1‐month‐delayed (*D* = −1) positive relationship between chlorophyll *a* concentration and sp. *V*_max_ of the three enzymatic activities (Figure 5A and Table S4), which indicates that shifts in chlorophyll *a* concentration precede changes in the sp. *V*_max_. A positive correlation was found between the *K*_m_ of the HA system of LAP and the LA system of βG, respectively. Conversely, cyanobacterial abundance always showed negative correlations with sp. *V*_max_ (Figure 5B and Table S5), which were contemporary for the two enzymatic systems of LAP and the HA system of αG and with a month of delay in the rest of the cases. Likewise, the correlations with the *K*_m_ were overall negative (Figure 5B and Table S5).

Remarkably, most of the correlations between the relative abundance of different bacterial groups and kinetic parameters did not indicate a time delay (Figure 5 and Tables S4 and S5). A positive correlation was found between *Bacteroidetes* and the kinetic parameters of LAP and αG, as well as with the HA specific *V*_max_ of βG. *Gammaproteobacteria* were positively associated with the kinetic parameters of glucosidases, while negative correlations were observed with LAP activity. Regarding *Alphaproteobacteria*, positive correlations were detected between the abundance of *Roseobacter* and the specific *V*_max_ of the three enzymatic activities, while a negative correlation was found with HA *K*_m_ glucosidases. SAR11 abundance presented negative correlations with the kinetic parameters of glucosidase enzymes and the specific *V*_max_ of LAP.

Although the detailed associations between the environmental variables are beyond the purpose of this study, it should be noted that we detected only positive relationships between the primary producers and the phylogenetic groups, for example, cyanobacterial abundance and SAR11 (Figure 5A and Table S4). In addition, we found only negative relationships both between the two phytoplanktonic groups and between the phylogenetic groups, for example, *Roseobacter* with *Gammaproteobacteria* and SAR11 (Figure 5B and Table S5). Altogether, these findings may point to the ability of certain members to proliferate under specific environmental conditions or may reflect niche‐ or resource–competitive interactions.

## DISCUSSION

### The importance of the determination of the kinetic parameters by computing multiphasic models

This study reports for the first time the seasonal and inter‐annual variation in the kinetic parameters of LAP and βG and αG in surface waters of a temperate coastal ecosystem. Hydrolysis rates of the three activities were fitted to models of increasing complexity, revealing that at least two different enzymatic systems, characterized by different substrate affinities, were present simultaneously for any of the enzymatic activities tested throughout the year. This result confirms that the occurrence of different isoenzymes acting on the same substrate reported in previous studies (Arrieta & Herndl, 2002; Vrba et al., 1996) is a common feature that persists throughout the year in coastal ecosystems.

Kinetic studies of the hydrolysis of HMW‐DOM in marine ecosystems by computing multiphasic models are scarce and restricted in scope. Previous studies have focused on of LAP and βG activities (Unanue et al., 1999; van Wambeke et al., 2021) and alkaline phosphatase activity (Bogé et al., 2012, 2013; van Wambeke et al., 2021) in surface waters or aminopeptidase activity in benthic waters and sediment samples (Talbot & Bianchi, 1997; Tholosan et al., 1999). In fact, most of the research on the EEAs in seawater is based on hydrolysis rates acquired by adding the substrate proxies in a unique saturating substrate concentration between 50 ans 500 μM (Baña et al., 2020; Misic et al., 2006). According to our results, this experimental approach misses an important part of the enzymatic response because it only allows the characterization of the LA enzymatic system. Consequently, the activity of the HA enzymatic systems, which prevail at natural concentrations of polymers in seawater, usually <5 μM in the case of proteins and polysaccharides (Keil & Kirchman, 1994; Nagata, 2008), is masked and remains poorly understood. In this sense, our findings highlight the importance of kinetic studies for the characterization of EEAs in natural samples since those based on a single saturating concentration may not adequately describe the dynamics of enzymatic activity at relevant substrate concentrations.

Our results show that a multiconcentration assay comprising at least 12 substrate concentrations allows reliable estimation of the kinetic parameters, minimizing the effect of data scattering and provide a minimal number of points for running complex models (Panikov et al., 1992). In the case of Armintza ecosystem, we acknowledge the importance of increasing the upper limit for MCA‐LAP concentration range to 600–1000 μM to refine the calculation of the LA kinetic parameters. In the case of the glucosidases, we should consider expanding the number of concentrations added in the low range below 40 μM for a better estimation of HA *K*_m_.

### The ecological relevance of HA and LA enzymatic systems

Natural microbial assemblages express a range of different hydrolases with different affinities and relative abundances that can only be assessed by separating each isoenzyme (Arrieta & Herndl, 2002). Our bulk kinetic approach cannot distinguish all of them separately, but nevertheless, it allows the detection of two prevalent classes of isoenzymes showing distinct substrate affinities representing the two extremes of the range of kinetic parameters. These two affinity classes give important clues about how the microbial communities react to changing substrate availability while using a simple and cost‐effective method.

The permanent detection of two distinct enzymatic systems at Armintza coastal station may reflect the transient HMW‐DOM pulses that occur in marine environments. Seawater is a highly diluted medium characterized by low concentrations of organic substrates, but also containing *hot‐spots* of highly concentrated materials like polymer gels (Verdugo, 2012), and biological particles such as decaying phytoplankton cells or zooplankton excreta (Middelboe et al., 1996; Moran et al., 2022; Myklestad, 2000; Simon et al., 2002; Thornton, 2014). These *hot‐spots* provide spatially heterogeneous microniches expanding the range of substrate concentration over several orders of magnitude as compared to bulk seawater (Amin et al., 2012; Stocker, 2012; Unanue et al., 1998).

In this context, the values of LA *K*_m_ of LAP (~100–1000 μM) and the glucosidases (~2–500 μM) may correspond to isoenzymes produced by those bacteria that inhabit the *hot‐spots*. In contrast, the values of the HA *K*_m_ (<10 μM for LAP and <5 μM for the glucosidases) could reflect the adaptations of free‐living bacteria to the low concentrations of polymeric substrates in the liquid phase (Unanue et al., 1999). Other authors have also attributed multiphasic kinetics to the coexistence of copiotrophic and oligotrophic microorganisms (Panikov et al., 1992).

### Enzymatic response of the bacterial community to environmental changes

The LSA method allowed us to find not only contemporary but also time‐delayed associations between variables, enabling extended interpretations of the dynamics of the enzymatic response of the bacterial community in the Armintza coastal ecosystem.

We interpret the local similarity correlations with the different primary producers as indicative of the liberation of organic matter that favours the secretion of a specific set of EEAs. In this sense, the positive association between chlorophyll *a* concentration and the specific *V*_max_ of both enzymatic systems of the three EEAs suggests that the development of the eukaryotic phytoplankton *bloom* triggers the secretion of enzymes with high hydrolytic capacity by the bacterial community, most likely associated to the collapse of the *bloom*, decaying algal cells release HMW molecules to the environment (Becker et al., 2014; Bidle & Azam, 2001). In the case of the LA specific *V*_max_, the correlation was tighter to glucosidases than to LAP, which is consistent with reported release of carbon‐enriched organic matter during the wax and wane of *blooms* dominated by eukaryotic phytoplankton (Alldredge et al., 1995; Cisternas‐Novoa et al., 2015; Villacorte et al., 2015; Wetz & Wheeler, 2007). Similarly, Teeling et al. (2016) detected a peak in the abundance of carbohydrate‐active enzymes during a diatom *bloom* in the North Sea and high levels of expression of components of the TonB‐dependent transporters which may mediate the transport of substrates >600–800 Da, while transporters for low‐molecular‐weight substrates were under‐expressed.

In contrast, the negative correlation between the cyanobacterial abundance and the kinetic parameters of the EEAs suggests that the organic matter derived from this phytoplankton group does not contain large concentrations of appropriate substrates. Several authors have observed that cyanobacterial exudates are enriched in low‐molecular‐weight organic molecules (Becker et al., 2014; Seymour et al., 2010), such as organic acids, that may comprise up to 20% of the dissolved organic carbon released (Bertilsson et al., 2003). Therefore, production enzymes with high hydrolytic capacity for the cleavage of HMW‐DOM may not be stimulated by cyanobacterial‐derived materials lacking suitable substrates.

Variations in the structure of the phytoplankton community are often followed by shifts in the bacterial community (Baña et al., 2020; Teeling et al., 2016), which, in turn, leads to changes in the diversity of the isoenzymes that are secreted (Arrieta & Herndl, 2002). The correlations obtained with the LSA method evidenced that members of *Bacteroidetes* are likely producers of LAP and glucosidase isoenzymes. Moreover, the positive correlations with the specific *V*_max_ and the LA *K*_m_ of LAP and αG, suggest an optimization of the hydrolysis at high concentrations of substrate. These findings indicate that *Bacteroidetes* can efficiently exploit the DOM released during the eukaryotic phytoplankton *bloom* and to proliferate until it becomes the dominant group of the bacterial community in summer (Baña et al., 2020), as it has been previously reported in other ecosystems (Amin et al., 2012; Riemann et al., 2000; Teeling et al., 2016). Indeed, members of this phylum are usually associated to particulate organic aggregates and play a key role in degradation of HMW‐DOM because of their ability to synthesize a large number of polymer‐degrading enzymes, including glucosidases and peptidases (Amin et al., 2012; Bidle & Azam, 2001; Fernández‐Gómez et al., 2013).

In the Armintza coastal station, *Gammaproteobacteria* are important members of the bacterial community between late spring and summer, although to a lesser extent than *Bacteroidetes*. The results of the correlations distinguish the members of *Gammaproteobacteria* as producers of glucosidases, rather than LAP, hinting to a metabolic specialization in using carbohydrates. Several studies have described the formation of transparent exopolymer particles by diatoms (Alldredge et al., 1995; Villacorte et al., 2015; Wetz & Wheeler, 2007), whose production reaches its maximum during the senescence of the *bloom* (Cisternas‐Novoa et al., 2015) and entails the secretion of sulphated heteropolysaccharides into the environment (Passow, 2002). This circumstance might favour the proliferation of those bacteria like *Gammaproteobacteria* or *Bacteroidetes* able to use the carbohydrates that accumulate during summer.

*Alphaproteobacteria*, *Roseobacter* and SAR11, showed remarkable differences in their hydrolytic response. The correlations obtained with *Roseobacter* suggest that the members of this group are producers of both HA and LA isoenzymes of LAP and glucosidase. Moreover, in the case of the HA system of αG and βG, the positive correlations with the specific *V*_max_ and the negative correlations with the K_m_ may reflect an optimization in the use of carbohydrates at low substrate concentrations. This functional versatility may allow *Roseobacter* members to sporadically peak between the months of February and April by taking advantage of the concentration gradients of organic matter generated by the eukaryotic phytoplankton *bloom*. Several studies have described a larger relative abundances of *Roseobacter* during spring *blooms*, mainly formed by diatoms (Alonso‐Gutiérrez et al., 2009; Amin et al., 2012; Teeling et al., 2016), and their ability to switch between ecological niches, that is, bulk phase and aggregates (Moran et al., 2007; Riemann et al., 2000), favoured by the possession of *quorum‐sensing* mechanisms (Gram et al., 2002).

Conversely, the negative correlations between the kinetic parameters of the EEAs and SAR11 indicate that use of HMW‐DOM is less important for this group. Additional support for this idea comes from the fact that SAR11 are usually related to the free‐living lifestyle, lack *quorum‐sensing* mechanisms for particle adhesion (Giovannoni et al., 2005) and are specialized in the uptake of monomers at low substrate concentrations (Alonso & Pernthaler, 2006). Fernández‐Gómez et al. (Fernández‐Gómez et al., 2013) quantified a low number of glucosidases and a relatively high number of peptidases in the genome of *Candidatus* Pelagibacter. These findings reveal an intrinsic limited metabolic capacity to exploit resource‐rich conditions, favouring the dominance of SAR11 in the bacterial community at the end of autumn, coinciding with the collapse of the cyanobacterial *bloom*. Our results confirm those of Sarmento et al. (Sarmento et al., 2016), who observed that the members of *Bacteroidetes* and *Gammaproteobacteria* were more specialized in the degradation of exudates derived from eukaryotic algae, whereas SAR11 was more specialized in using organic matter derived from *Synechococcus*.

## CONCLUSION

Determination of the kinetic parameters of EEAs under varying environmental situations is of great interest since it provides information about the adaptation of the bacterial community in the use of available organic matter, uncovering patterns that would not be detectable in single, saturating concentration studies. The analysis of the correlative ecological network of Armintza station unveiled an association between the organic matter released by the different phytoplanktonic groups and the secretion of EEAs by specific phylogenetic groups of the bacterial community. The springtime eukaryotic *bloom* triggers an increase in the availability of HMW‐DOM and selects those phylotypes that synthesize isoenzymes responsive to high concentrations of substrate, including members of *Bacteroidetes*, *Gammaproteobacteria* and, sporadically, *Roseobacter*. Conversely, the cyanobacterial *bloom* that occurs during summer‐autumn seems to release low molecular weight compounds, which favours the proliferation of members with a lower hydrolytic capacity, as is the case for SAR11.

## FUNDING INFORMATION

This work has been supported by projects EFICIENCIA (CTM2006‐08023) and CAMBIO (CTM2010‐19308), co‐financed by Ministry of Science and Innovation of the Spanish Government and European FEDER founds, the Basque Government (Grant to Research Group IT1657‐22) and by the UPV/EHU (Grant to Research Group GIU10/17). Naiara Abad was supported by a scholarship from the Basque Government and currently by the grant Margarita Salas from the European Union—NextGenerationEU through the UPV/EHU. Zuriñe Baña and Ainhoa Uranga were financed by scholarships from the UPV/EHU.

## CONFLICT OF INTEREST

The authors declare that the research was conducted in the absence of any commercial or financial relationships that could be construed as a potential conflict of interest.

## Supporting information


**Figure S1.** Representation of the four models used in this study

**Figure S2.** Box–whisker plots of the environmental variables of the ecosystem

**Table S1.** Goodness of data fits to each of the models

**Table S2.** Values of the Michaelis half‐saturation constant (*K*_m_, μM), the maximum hydrolysis rate of the enzyme reaction (*V*_max_, nM·h^−1^) and the cell‐specificic maximum hydrolysis rate (sp. *V*_max_, amol·cell^−1^·h^−1^)

**Table S3.** Values of the Michaelis half‐saturation constant (*K*_m_, μM), the maximum hydrolysis rate of the enzyme reaction (*V*_max_, nM·h^−1^) and the cell‐specificic maximum hydrolysis rate (sp. *V*_max_, amol·cell^−1^·h^−1^)

**Table S4.** Summary of the highest LS scores found by the local similarity analysis from the positive correlations

**Table S5.** Summary of the highest LS scores found by the local similarity analysis from the negative correlations

## Data Availability

The supplemental dataset available at https://doi.org/10.5281/zenodo.6868359 provides the code developed for the determination of the kinetic parameters of the EEAs and an updated version of the code developed by Ruan et al. (2006) used to conduct the local similarity analysis in this research work.
