# Supplementary figures and images for: Kinetic modulation of bacterial hydrolases by microbial community structure in coastal waters
Source: Environ Microbiol. 2022 Dec 19;25(2):548–61. doi: 10.1111/1462-2920.16297 (PMC10108013; doi:10.1111/1462-2920.16297)

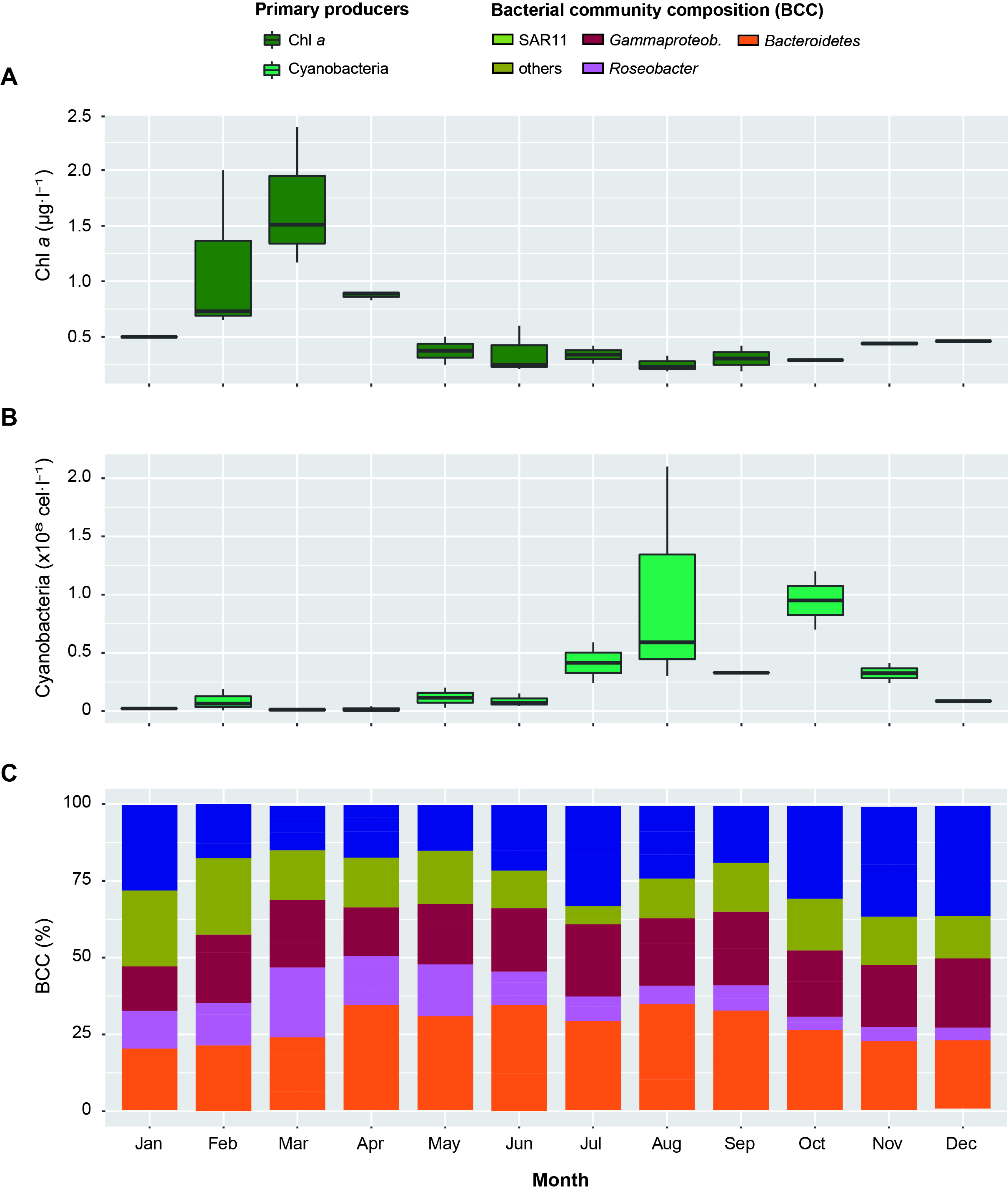

Supplement: Supplementary file 1 — Figure S1. Representation of the four models used in this study [file EMI-25-548-s004.tif]

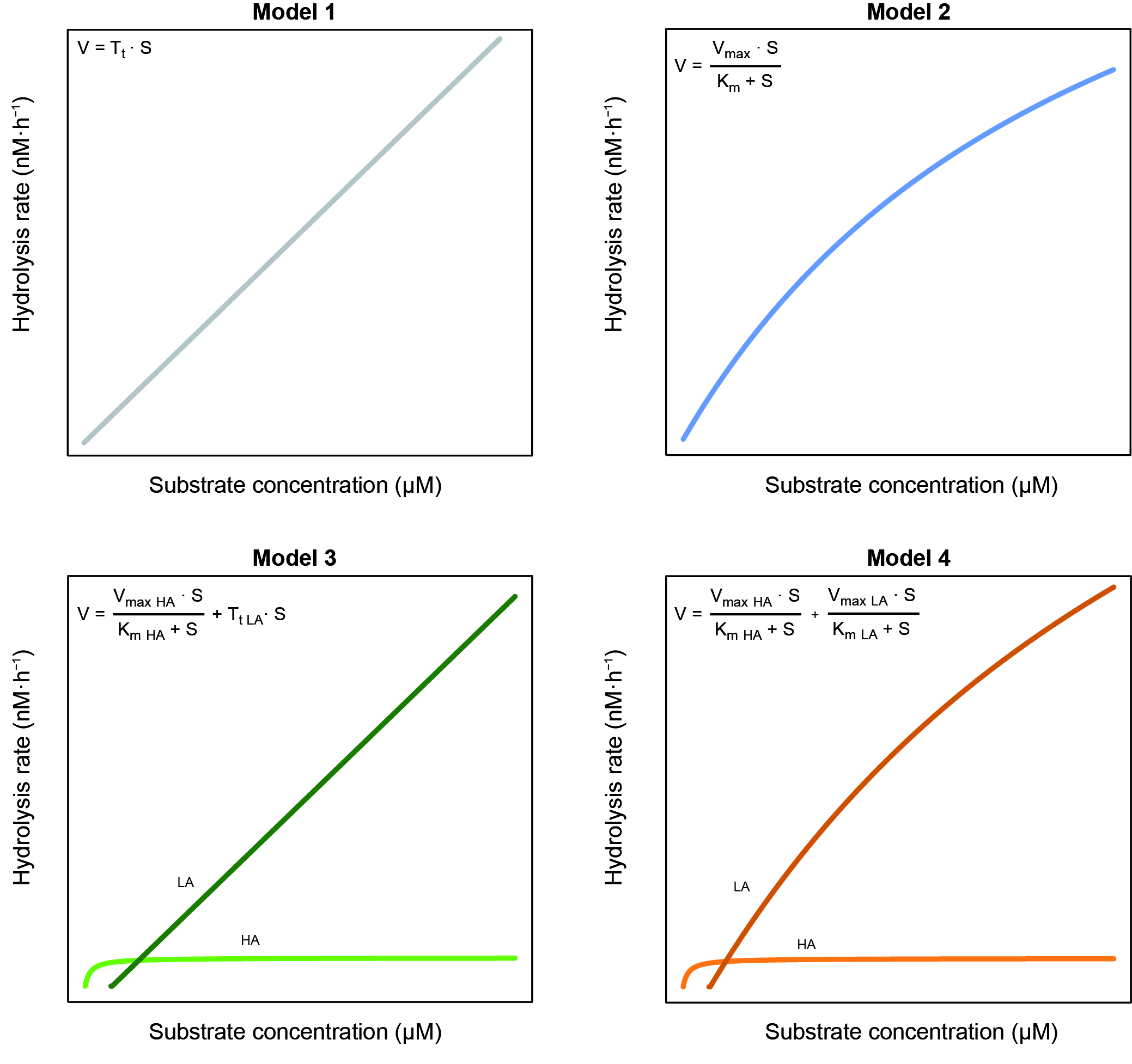

Supplement: Supplementary file 2 — Figure S2. Box–whisker plots of the environmental variables of the ecosystem [file EMI-25-548-s003.tif]
