# Supplementary material for: Kinetic modulation of bacterial hydrolases by microbial community structure in coastal waters
Source: Environ Microbiol. 2022 Dec 19;25(2):548–61. doi: 10.1111/1462-2920.16297 (PMC10108013; doi:10.1111/1462-2920.16297)
Supplement: Supplementary file 3 — Table S1. Goodness of data fits to each of the models [file EMI-25-548-s006.docx]

| **Supplementary table ST1.** Goodness of data fits to each of the models obtained using the corrected Akaike information criterion (AICc). The model considered for subsequent analysis has been highlighted with a grey background. | | | | | | | | | | | | |
| --- | --- | --- | --- | --- | --- | --- | --- | --- | --- | --- | --- | --- |
| Sample | Leucine aminopeptidase | | | | β-glucosidase | | | | α-glucosidase | | | |
|  | M1 | M2 | M3 | M4 | M1 | M2 | M3 | M4 | M1 | M2 | M3 | M4 |
| Feb11 | 310 | n.c. | 301 | 259 | 64 | 11 | -4 | -30 | 73 | 54 | 56 | 35 |
| Mar11 | 343 | 268 | n.c. | 262 | 8 | -25 | -13 | -49 | 68 | 7 | 48 | -70 |
| Apr11 | 410 | 333 | 402 | 251 | 114 | 28 | 101 | -17 | 95 | 21 | 76 | -26 |
| May11 | 327 | 264 | n.c. | 165 | 115 | 53 | 97 | 16 | -8 | n.c. | -26 | -39 |
| Jun11 | n.c. | 287 | 363 | 240 | 78 | 14 | 57 | -55 | 47 | 13 | 5 | -76 |
| Aug11 | 264 | n.c. | 267 | 239 | -59 | -112 | -68 | -171 | -67 | -120 | -86 | -140 |
| Sep11 | 265 | n.c. | 256 | 205 | -8 | -78 | -29 | -114 | 0 | -45 | -11 | -50 |
| Oct11 | 313 | n.c. | 314 | 268 | -8 | -28 | -8 | -20 | -50 | -106 | -70 | -121 |
| Nov11 | 319 | n.c. | 323 | 281 | -28 | -104 | -28 | -99 | -105 | n.c. | -160 | -152 |
| Jan12 | 261 | n.c. | 254 | 217 | -33 | -147 | -38 | -167 | -57 | -111 | -70 | -121 |
| Feb12 | 310 | n.c. | 311 | 266 | -52 | -128 | -61 | -183 | -113 | -167 | -151 | -195 |
| Mar12 | 366 | 282 | 354 | 270 | 40 | -36 | 36 | -53 | -31 | -44 | -58 | -61 |
| Apr12 | 402 | 272 | 405 | 282 | 88 | 34 | 66 | -37 | 51 | 13 | 21 | -83 |
| May12 | 371 | n.c. | 370 | 302 | 49 | -36 | 31 | -146 | 44 | -3 | 15 | -53 |
| Jun12 | 440 | 380 | n.c. | 356 | 115 | 13 | 81 | -70 | n.d. | n.d. | n.d. | n.d. |
| Jul12 | 311 | n.c. | 307 | 245 | 22 | -96 | 13 | -134 | 20 | -29 | -7 | -65 |
| Aug12 | 293 | n.c. | 286 | 268 | 23 | -104 | 18 | -143 | -32 | -67 | -42 | -70 |
| Oct12 | 329 | n.c. | 330 | 283 | -14 | -151 | -57 | -164 | -23 | -41 | -31 | -55 |
| Nov12 | 351 | n.c. | 353 | 296 | -29 | -113 | -75 | -166 | -8 | -39 | n.c. | -95 |
| Dec12 | 339 | n.c. | 343 | 305 | -63 | -80 | -90 | -97 | -33 | -76 | -38 | -96 |
| Feb13 | 302 | n.c. | 300 | 251 | 32 | 16 | -21 | -48 | 10 | -73 | -34 | -109 |
| Mar13 | 286 | n.c. | 276 | 256 | 11 | -46 | -13 | -107 | 13 | -62 | 9 | -94 |
| Apr13 | 354 | n.c. | 335 | 258 | 33 | -20 | -14 | -126 | -6 | -91 | -53 | -146 |
| Jun13 | 425 | 335 | 413 | 260 | 94 | 8 | 76 | -80 | -18 | n.c. | -53 | -50 |
| Jul13 | n.d. | n.d. | n.d. | n.d. | 56 | -41 | 45 | -169 | -80 | n.c. | -91 | -84 |
| Aug13 | 268 | n.c. | 263 | 157 | 70 | 2 | 48 | -55 | -34 | -41 | -47 | -48 |
| Sep13 | 303 | n.c. | 301 | 270 | -62 | -118 | -87 | -140 | -101 | n.c. | -128 | -131 |
| M: model; n.c.: no convergence; n.d.: no data. | | | | | | | | | | | | |
