# Supplementary material for: Kinetic modulation of bacterial hydrolases by microbial community structure in coastal waters
Source: Environ Microbiol. 2022 Dec 19;25(2):548–61. doi: 10.1111/1462-2920.16297 (PMC10108013; doi:10.1111/1462-2920.16297)
Supplement: Supplementary file 4 — Table S2. Values of the Michaelis half‐saturation constant (K m, μM), the maximum hydrolysis rate of the enzyme reaction (V max, nM·h−1) and the cell‐specificic maximum hydrolysis rate (sp. V max, amol·cell−1·h−1) [file EMI-25-548-s005.docx]

| **Supplementary table ST2.** Values of the Michaelis half-saturation constant (K_m_, µM), the maximum hydrolysis rate of the enzyme reaction (V_max_, nM·h^-1^) and the cell-specificic maximum hydrolysis rate (sp. V_max_, amol·cell^-1^·h^-1^) of leucine aminopeptidase activity obtained throughout the interannual study. | | | | | | | |
| --- | --- | --- | --- | --- | --- | --- | --- |
| Sample | Leucine aminopeptidase | | | | | | |
|  | High-affinity system | | |  | Low-affinity system | | |
|  | K_m_ | V_max_ | sp. V_max_ |  | K_m_ | V_max_ | sp. V_max_ |
| Feb11 | 1.5 | 6.3 | 24 |  | 692 | 283 | 1083 |
| Mar11 | 2.7 | 14.3 | 28 |  | 260 | 183 | 363 |
| Apr11 | 6.9 | 27.2 | 30 |  | 597 | 403 | 450 |
| May11 | 1.3 | 10.1 | 11 |  | 757 | 299 | 321 |
| Jun11 | 0.9 | 7.7 | 8 |  | 592 | 379 | 387 |
| Aug11 | 1.4 | 3.0 | 3 |  | 432 | 239 | 260 |
| Sep11 | 4.7 | 10.7 | 5 |  | 446 | 243 | 116 |
| Oct11 | 4.3 | 11.0 | 14 |  | 411 | 274 | 356 |
| Nov11 | 4.0 | 10.1 | 12 |  | 295 | 243 | 278 |
| Jan12 | 7.0 | 11.4 | 12 |  | 460 | 236 | 242 |
| Feb12 | 4.4 | 9.2 | 6 |  | 403 | 255 | 161 |
| Mar12 | 9.6 | 33.7 | 29 |  | 525 | 405 | 353 |
| Apr12 | 7.4 | 17.7 | 22 |  | 224 | 195 | 237 |
| May12 | 7.2 | 19.7 | 90 |  | 221 | 202 | 922 |
| Jun12 | 1.6 | 22.9 | 19 |  | 379 | 348 | 288 |
| Jul12 | 3.8 | 14.5 | 12 |  | 715 | 432 | 345 |
| Aug12 | 7.7 | 16.2 | 16 |  | 898 | 452 | 446 |
| Oct12 | 4.9 | 12.1 | 10 |  | 373 | 276 | 229 |
| Nov12 | 6.0 | 12.3 | 14 |  | 251 | 248 | 273 |
| Dec12 | 4.0 | 8.5 | 12 |  | 215 | 175 | 247 |
| Feb13 | 5.1 | 10.1 | 12 |  | 482 | 247 | 291 |
| Mar13 | 5.9 | 10.3 | 11 |  | 617 | 293 | 323 |
| Apr13 | 6.0 | 19.2 | 34 |  | 660 | 340 | 603 |
| Jun13 | 4.9 | 27.1 | 46 |  | 143 | 187 | 315 |
| Jul13 | n.d. | n.d. | n.d. |  | n.d. | n.d. | n.d. |
| Aug13 | 1.1 | 2.7 | 2 |  | 983 | 263 | 221 |
| Sep13 | 10.5 | 6.5 | 6 |  | 315 | 190 | 172 |
| n.d.: no data. | | | | | | | |
