# Supplementary material for: Kinetic modulation of bacterial hydrolases by microbial community structure in coastal waters
Source: Environ Microbiol. 2022 Dec 19;25(2):548–61. doi: 10.1111/1462-2920.16297 (PMC10108013; doi:10.1111/1462-2920.16297)
Supplement: Supplementary file 5 — Table S3. Values of the Michaelis half‐saturation constant (K m, μM), the maximum hydrolysis rate of the enzyme reaction (V max, nM·h−1) and the cell‐specificic maximum hydrolysis rate (sp. V max, amol·cell−1·h−1) [file EMI-25-548-s001.docx]

| **Supplementary Table ST3.** Values of the Michaelis half-saturation constant (K_m_, µM), the maximum hydrolysis rate of the enzyme reaction (V_max_, nM·h^-1^) and the cell-specificic maximum hydrolysis rate (sp. V_max_, amol·cell^-1^·h^-1^) of β- and α-glucosidase activities obtained throughout the interannual study. | | | | | | | | | | | | | |
| --- | --- | --- | --- | --- | --- | --- | --- | --- | --- | --- | --- | --- | --- |
| Sample | β-glucosidase | | | | | |  | α-glucosidase | | | | | |
|  | High-affinity system | | | Low-affinity system | | |  | High-affinity system | | | Low-affinity system | | |
|  | K_m_ | V_max_ | sp. V_max_ | K_m_ | V_max_ | sp. V_max_ |  | K_m_ | V_max_ | sp. V_max_ | K_m_ | V_max_ | sp. V_max_ |
| Feb11 | 0.10 | 0.64 | 2.44 | 57 | 1.86 | 7.12 |  | 0.54 | 0.54 | 2.05 | 126 | 3.01 | 11.50 |
| Mar11 | 0.57 | 0.39 | 0.77 | 135 | 3.94 | 7.81 |  | 0.25 | 0.33 | 0.65 | 103 | 2.15 | 4.27 |
| Apr11 | 3.01 | 0.98 | 1.09 | 261 | 8.40 | 9.37 |  | 5.87 | 0.66 | 0.73 | 198 | 4.86 | 5.42 |
| May11 | 1.29 | 0.88 | 0.95 | 267 | 7.78 | 8.36 |  | 1.53 | 0.36 | 0.39 | 486 | 6.48 | 6.95 |
| Jun11 | 0.04 | 0.41 | 0.42 | 163 | 3.80 | 3.87 |  | 0.39 | 0.53 | 0.54 | 187 | 4.37 | 4.45 |
| Aug11 | 0.33 | 0.10 | 0.11 | 437 | 2.04 | 2.22 |  | 0.16 | 0.09 | 0.10 | 45 | 0.46 | 0.50 |
| Sep11 | 1.09 | 0.35 | 0.17 | 28 | 1.03 | 0.49 |  | 3.77 | 0.50 | 0.24 | 68 | 1.61 | 0.77 |
| Oct11 | n.d. | n.d. | n.d. | 187 | 2.70 | 3.51 |  | 0.59 | 0.15 | 0.19 | 46 | 0.69 | 0.90 |
| Nov11 | n.d. | n.d. | n.d. | 20 | 0.48 | 0.54 |  | 2.29 | 0.17 | 0.19 | n.d. | n.d. | n.d. |
| Jan12 | 0.04 | 0.04 | 0.04 | 45 | 0.72 | 0.74 |  | 0.04 | 0.04 | 0.04 | 45 | 0.72 | 0.74 |
| Feb12 | 0.32 | 0.11 | 0.07 | 99 | 0.56 | 0.35 |  | 0.40 | 0.05 | 0.03 | 31 | 0.20 | 0.13 |
| Mar12 | 0.23 | 0.21 | 0.18 | 57 | 1.53 | 1.33 |  | 0.33 | 0.26 | 0.22 | 119 | 1.32 | 1.15 |
| Apr12 | 0.26 | 0.68 | 0.82 | 398 | 4.61 | 5.59 |  | 0.12 | 0.40 | 0.48 | 447 | 8.08 | 9.81 |
| May12 | 0.54 | 0.44 | 2.01 | 116 | 1.46 | 6.68 |  | 0.52 | 0.50 | 2.28 | 75 | 1.99 | 9.07 |
| Jun12 | 0.07 | 1.06 | 0.88 | 9 | 2.20 | 1.82 |  | n.d. | n.d. | n.d. | n.d. | n.d. | n.d. |
| Jul12 | 0.50 | 0.28 | 0.22 | 24 | 0.72 | 0.57 |  | 0.73 | 0.41 | 0.33 | 100 | 2.01 | 1.60 |
| Aug12 | 0.85 | 0.21 | 0.21 | 43 | 0.99 | 0.98 |  | 5.46 | 0.30 | 0.30 | 136 | 1.36 | 1.34 |
| Oct12 | 0.45 | 0.30 | 0.25 | 2 | 0.40 | 0.33 |  | 1.28 | 0.23 | 0.19 | 327 | 3.20 | 2.66 |
| Nov12 | 0.03 | 0.19 | 0.21 | 10 | 0.39 | 0.43 |  | 0.38 | 0.30 | 0.33 | 153 | 1.73 | 1.90 |
| Dec12 | 2.16 | 0.20 | 0.28 | 279 | 1.47 | 2.06 |  | 3.19 | 0.26 | 0.37 | 57 | 1.34 | 1.88 |
| Feb13 | 0.30 | 0.51 | 0.61 | 407 | 5.17 | 6.11 |  | 0.09 | 0.32 | 0.38 | 12 | 0.65 | 0.77 |
| Mar13 | 0.86 | 0.29 | 0.32 | 481 | 6.21 | 6.83 |  | 1.25 | 0.24 | 0.26 | 298 | 4.60 | 5.06 |
| Apr13 | 0.64 | 0.46 | 0.81 | 301 | 6.68 | 11.84 |  | 0.16 | 0.30 | 0.53 | 18 | 0.64 | 1.13 |
| Jun13 | 0.64 | 0.67 | 1.13 | 148 | 2.84 | 4.77 |  | 2.19 | 0.37 | 0.63 | 265 | 2.37 | 3.98 |
| Jul13 | 0.27 | 0.33 | 0.23 | 128 | 2.79 | 2.01 |  | 0.96 | 0.15 | 0.11 | 367 | 2.48 | 1.79 |
| Aug13 | 0.40 | 0.66 | 0.56 | 23 | 1.80 | 1.52 |  | 2.59 | 0.25 | 0.21 | 118 | 0.96 | 0.81 |
| Sep13 | 2.90 | 0.25 | 0.23 | 87 | 1.17 | 1.06 |  | 1.34 | 0.15 | 0.14 | 244 | 1.38 | 1.25 |
| n.d.: no data. | | | | | | | | | | | | | |
