# Supplementary material for: Kinetic modulation of bacterial hydrolases by microbial community structure in coastal waters
Source: Environ Microbiol. 2022 Dec 19;25(2):548–61. doi: 10.1111/1462-2920.16297 (PMC10108013; doi:10.1111/1462-2920.16297)
Supplement: Supplementary file 6 — Table S4. Summary of the highest LS scores found by the local similarity analysis from the positive correlations [file EMI-25-548-s002.docx]

| **Supplementary Table ST4.** Summary of the highest LS scores found by the Local Similarity Analysis (Ruan *et al.*, 2006) from the positive correlations between all the variables analysed in this study. The subscripts HA and LA stand for the high-affinity and low-affinity enzymatic systems. The columns in succession are: X (factor 1), Y (factor 2), LS (local similarity score), sX (start of the best alignment in the sequence of X factor), sY (start of the best alignment in the sequence of Y factor), Len (alignment length), D (shift of the Y factor sequence compared to the X factor sequence, -: X is ahead of Y, + otherwise), P-val (*p*-value for the LS score), Q-val (*q-*value calculated for P-val). | | | | | | | | |  |
| --- | --- | --- | --- | --- | --- | --- | --- | --- | --- |
|  |  |  |  |  |  |  |  |  |  |
|  |  |  |  |  |  |  |  |  |  |
|  |  |  |  |  |  |  |  |  |  |
| X | Y | LS | sX | sY | Len | D | P-val | Q-val |  |
| *Kinetic parameter vs Kinetic parameter* | | | | | | | | |  |
| LAP K_m HA_ | LAP sp. V_max HA_ | 0.488 | 3 | 3 | 29 | 0 | 0.001 | 0.019 |  |
| βG K_m HA_ | βG sp. V_max HA_ | 0.355 | 2 | 3 | 26 | -1 | 0.005 | 0.029 |  |
| αG sp. V_max LA_ | αG K_m LA_ | 0.571 | 1 | 1 | 31 | 0 | 0.001 | 0.011 |  |
| βG sp. V_max LA_ | βG K_m LA_ | 0.565 | 2 | 2 | 31 | 0 | 0.001 | 0.012 |  |
| LAP sp. V_max LA_ | αG sp. V_max LA_ | 0.411 | 1 | 1 | 32 | 0 | 0.004 | 0.016 |  |
| LAP sp. V_max LA_ | βG sp. V_max LA_ | 0.495 | 1 | 1 | 32 | 0 | 0.003 | 0.014 |  |
| βG sp. V_max LA_ | αG sp. V_max LA_ | 0.502 | 1 | 1 | 32 | 0 | 0.002 | 0.005 |  |
| LAP sp. V_max_ _HA_ | αG sp. V_max HA_ | 0.546 | 1 | 1 | 32 | 0 | 0.001 | 0.007 |  |
| LAP sp. V_max_ _HA_ | βG sp. V_max HA_ | 0.504 | 1 | 1 | 29 | 0 | 0.001 | 0.007 |  |
| βG sp. V_max HA_ | αG sp. V_max HA_ | 0.715 | 1 | 1 | 32 | 0 | 0.001 | 0.007 |  |
| LAP sp. V_max_ _HA_ | LAP sp. V_max LA_ | 0.550 | 1 | 1 | 32 | 0 | 0.001 | 0.007 |  |
| αG sp. V_max HA_ | αG sp. V_max LA_ | 0.632 | 1 | 1 | 32 | 0 | 0.001 | 0.007 |  |
| βG sp. V_max HA_ | βG sp. V_max LA_ | 0.567 | 1 | 1 | 32 | 0 | 0.001 | 0.007 |  |
| αG K_m HA_ | βG K_m HA_ | 0.570 | 1 | 1 | 26 | 0 | 0.001 | 0.008 |  |
| *Variable vs Kinetic parameter* | | | | | | | | |  |
| chl *a* | αG sp. V_max LA_ | 0.345 | 1 | 2 | 18 | -1 | 0.001 | 0.008 |  |
| chl *a* | αG sp. V_max HA_ | 0.371 | 1 | 2 | 31 | -1 | 0.005 | 0.021 |  |
| chl *a* | βG sp. V_max LA_ | 0.516 | 1 | 2 | 31 | -1 | 0.002 | 0.012 |  |
| chl *a* | βG sp. V_max HA_ | 0.377 | 1 | 2 | 28 | -1 | 0.006 | 0.024 |  |
| chl *a* | LAP sp. V_max LA_ | 0.397 | 1 | 2 | 31 | -1 | 0.005 | 0.021 |  |
| chl *a* | LAP sp. V_max_ _HA_ | 0.372 | 1 | 2 | 31 | -1 | 0.008 | 0.029 |  |
| chl *a* | βG K_m LA_ | 0.478 | 7 | 8 | 25 | -1 | 0.001 | 0.037 |  |
| chl *a* | LAP K_m HA_ | 0.390 | 3 | 3 | 29 | 0 | 0.008 | 0.038 |  |
| cyan | αG K_m HA_ | 0.502 | 1 | 1 | 32 | 0 | 0.001 | 0.025 |  |
| btrd | LAP sp. V_max_ _HA_ | 0.408 | 3 | 3 | 27 | 0 | 0.005 | 0.047 |  |
| btrd | LAP sp. V_max LA_ | 0.418 | 2 | 2 | 28 | 0 | 0.003 | 0.030 |  |
| btrd | βG sp. V_max HA_ | 0.396 | 1 | 1 | 29 | 0 | 0.003 | 0.026 |  |
| btrd | αG sp. V_max HA_ | 0.421 | 1 | 1 | 28 | 0 | 0.002 | 0.023 |  |
| btrd | αG sp. V_max LA_ | 0.483 | 1 | 2 | 28 | -1 | 0.002 | 0.025 |  |
| btrd | LAP K_m LA_ | 0.416 | 3 | 3 | 23 | 0 | 0.004 | 0.050 |  |
| btrd | αG K_m LA_ | 0.482 | 3 | 4 | 29 | -1 | 0.001 | 0.039 |  |
| sar11 | αG K_m HA_ | 0.450 | 1 | 1 | 27 | 0 | 0.001 | 0.019 |  |
| ros | LAP sp. V_max HA_ | 0.416 | 1 | 1 | 32 | 0 | 0.002 | 0.029 |  |
| ros | LAP sp. V_max LA_ | 0.418 | 1 | 1 | 32 | 0 | 0.004 | 0.038 |  |
| ros | βG sp. V_max HA_ | 0.348 | 1 | 1 | 30 | 0 | 0.009 | 0.050 |  |
| ros | βG sp. V_max LA_ | 0.395 | 1 | 1 | 32 | 0 | 0.006 | 0.050 |  |
| ros | αG sp. V_max HA_ | 0.369 | 1 | 1 | 32 | 0 | 0.007 | 0.050 |  |
| ros | αG sp. V_max LA_ | 0.372 | 1 | 1 | 32 | 0 | 0.016 | 0.049 |  |
| **Supplementary Table ST4 (continued)** | | | | | | | | | |
| X | Y | LS | sX | sY | Len | D | P-val | Q-val |  |
| ros | βG K_m LA_ | 0.411 | 8 | 8 | 25 | 0 | 0.003 | 0.046 |  |
| gam | βG sp. V_max LA_ | 0.323 | 1 | 1 | 26 | 0 | 0.010 | 0.042 |  |
| gam | αG sp. V_max LA_ | 0.422 | 1 | 1 | 27 | 0 | 0.002 | 0.021 |  |
| gam | βG K_m HA_ | 0.401 | 7 | 7 | 20 | 0 | 0.004 | 0.023 |  |
| gam | αG K_m LA_ | 0.397 | 3 | 3 | 28 | 0 | 0.006 | 0.048 |  |
| *Variable vs Variable* | | | | | | | | |  |
| ros | chl *a* | 0.497 | 2 | 1 | 31 | 1 | 0.001 | 0.002 |  |
| cyan | sar11 | 0.431 | 1 | 1 | 31 | 0 | 0.001 | 0.037 |  |
| gam | cyan | 0.395 | 2 | 2 | 29 | 0 | 0.009 | 0.044 |  |
| LAP: leucine aminopeptidase; βG: β-glucosidase; αG: α-glucosidase; chl*a*: chlorophyll *a* concentration (µg·l^-1^); cyan: cyanobacterial abundance (10^8^ cell·l^-1^); btrd: *Bacteroidetes* (%); sar11: SAR11 (%); ros: *Roseobacter* and members of SAR83 (%); gam: *Gammaproteobacteria* (%). | | | | | | | | |  |
