# Supplementary material for: Kinetic modulation of bacterial hydrolases by microbial community structure in coastal waters
Source: Environ Microbiol. 2022 Dec 19;25(2):548–61. doi: 10.1111/1462-2920.16297 (PMC10108013; doi:10.1111/1462-2920.16297)
Supplement: Supplementary file 7 — Table S5. Summary of the highest LS scores found by the local similarity analysis from the negative correlations [file EMI-25-548-s007.docx]

| **Supplementary Table ST5.** Summary of the highest LS scores found by the Local Similarity Analysis (Ruan *et al.*, 2006) from the negative correlations between all the variables analysed in this study. The subscripts HA and LA stand for the high-affinity and low-affinity enzymatic systems. The columns in succession are: X (factor 1), Y (factor 2), LS (local similarity score), sX (start of the best alignment in the sequence of X factor), sY (start of the best alignment in the sequence of Y factor), Len (alignment length), D (shift of the Y factor sequence compared to the X factor sequence, -: X is ahead of Y, + otherwise), P-val (*p*-value for the LS score), Q-val (*q-*value calculated for P-val). | | | | | | | | |  |
| --- | --- | --- | --- | --- | --- | --- | --- | --- | --- |
|  |  |  |  |  |  |  |  |  |  |
|  |  |  |  |  |  |  |  |  |  |
|  |  |  |  |  |  |  |  |  |  |
| **X** | **Y** | **LS** | **sX** | **sY** | **Len** | **D** | **P-val** | **Q-val** |  |
| *Variable vs Kinetic parameter* | | | | | | | | |  |
| chl *a* | αG K_m HA_ | 0.381 | 6 | 7 | 26 | -1 | 0.006 | 0.012 |  |
| cyan | αG sp. V_max_ _LA_ | 0.359 | 1 | 2 | 31 | -1 | 0.014 | 0.041 |  |
| cyan | αG sp. V_max HA_ | 0.435 | 1 | 1 | 32 | 0 | 0.003 | 0.016 |  |
| cyan | βG sp. V_max LA_ | 0.509 | 1 | 2 | 31 | -1 | 0.002 | 0.014 |  |
| cyan | βG sp. V_max HA_ | 0.372 | 1 | 2 | 31 | -1 | 0.012 | 0.039 |  |
| cyan | LAP sp. V_max LA_ | 0.476 | 1 | 1 | 32 | 0 | 0.001 | 0.009 |  |
| cyan | LAP sp. V_max_ _HA_ | 0.499 | 1 | 1 | 32 | 0 | 0.001 | 0.009 |  |
| cyan | LAP K_m HA_ | 0.493 | 2 | 2 | 29 | 0 | 0.001 | 0.014 |  |
| cyan | βG K_m LA_ | 0.486 | 8 | 8 | 24 | 0 | 0.002 | 0.042 |  |
| cyan | αG K_m LA_ | 0.352 | 2 | 3 | 17 | -1 | 0.013 | 0.037 |  |
| sar11 | LAP sp. V_max_ _HA_ | 0.463 | 1 | 2 | 31 | -1 | 0.001 | 0.021 |  |
| sar11 | LAP sp. V_max LA_ | 0.434 | 1 | 1 | 32 | 0 | 0.002 | 0.050 |  |
| sar11 | βG sp. V_max HA_ | 0.358 | 1 | 1 | 30 | 0 | 0.006 | 0.050 |  |
| sar11 | βG sp. V_max LA_ | 0.432 | 1 | 1 | 32 | 0 | 0.001 | 0.020 |  |
| sar11 | αG sp. V_max HA_ | 0.453 | 1 | 1 | 31 | 0 | 0.002 | 0.043 |  |
| sar11 | αG sp. V_max LA_ | 0.407 | 1 | 1 | 32 | 0 | 0.003 | 0.010 |  |
| sar11 | βG K_m LA_ | 0.396 | 10 | 10 | 23 | 0 | 0.007 | 0.003 |  |
| sar11 | αG K_m LA_ | 0.464 | 4 | 5 | 28 | -1 | 0.001 | 0.004 |  |
| ros | βG K_m HA_ | 0.309 | 8 | 8 | 25 | 0 | 0.048 | 0.021 |  |
| ros | αG K_m HA_ | 0.385 | 1 | 1 | 32 | 0 | 0.010 | 0.041 |  |
| gam | LAP sp. V_max_ _HA_ | 0.466 | 3 | 3 | 30 | 0 | 0.002 | 0.029 |  |
| gam | LAP sp. V_max LA_ | 0.489 | 4 | 4 | 27 | 0 | 0.001 | 0.030 |  |
| gam | LAP K_m HA_ | 0.467 | 3 | 3 | 32 | 0 | 0.001 | 0.049 |  |
| *Variable vs Variable* | | | | | | | | |  |
| chl *a* | cyan | 0.561 | 1 | 2 | 31 | -1 | 0.001 | 0.004 |  |
| chl *a* | btrd | 0.304 | 1 | 1 | 32 | 0 | 0.043 | 0.031 |  |
| chl *a* | sar11 | 0.440 | 1 | 2 | 31 | -1 | 0.001 | 0.006 |  |
| ros | cyan | 0.588 | 1 | 1 | 32 | 0 | 0.001 | 0.005 |  |
| btrd | sar11 | 0.455 | 4 | 4 | 28 | 0 | 0.004 | 0.016 |  |
| ros | sar11 | 0.584 | 1 | 1 | 32 | 0 | 0.001 | 0.001 |  |
| gam | ros | 0.331 | 2 | 2 | 30 | 0 | 0.032 | 0.028 |  |
| LAP: leucine aminopeptidase; βG: β-glucosidase; αG: α-glucosidase; chl*a*: chlorophyll *a* concentration (µg·l^-1^); cyan: cyanobacterial abundance (10^8^ cell·l^-1^); btrd: *Bacteroidetes* (%); sar11: SAR11 (%); ros: *Roseobacter* and members of SAR83 (%); gam: *Gammaproteobacteria* (%). | | | | | | | | | |
